# IL‐10 alleviates lipopolysaccharide‐induced skin scarring via IL‐10R/STAT3 axis regulating TLR4/NF‐κB pathway in dermal fibroblasts

**DOI:** 10.1111/jcmm.16250

**Published:** 2021-01-07

**Authors:** Jihong Shi, Shan Shi, Wenbo Xie, Ming Zhao, Yan Li, Jian Zhang, Na Li, Xiaozhi Bai, Weixia Cai, Xiaolong Hu, Dahai Hu, Juntao Han, Hao Guan

**Affiliations:** ^1^ Department of Burns and Cutaneous Surgery Xijing Hospital Fourth Military Medical University Xi'an China; ^2^ Queen Mary School Nanchang University Nanchang China

**Keywords:** dermal fibroblast, fibrosis, hypertrophic scar, inflammation, interleukin‐10, lipopolysaccharide, scar formation

## Abstract

Hypertrophic scar (HS) is a severe fibrotic skin disease. It has always been a major problem in clinical treatment, mainly because its pathogenesis has not been well understood. The roles of bacterial contamination and prolonged wound inflammation were considered significant. IL‐10 is a potent anti‐inflammatory cytokine and plays a pivotal role in wound healing and scar formation. Here, we investigate whether IL‐10 alleviates lipopolysaccharide (LPS)‐induced inflammatory response and skin scarring and explore the possible mechanism of scar formation. Our results showed that the expression of TLR4 and pp65 was higher in HS and HS‐derived fibroblasts (HSFs) than their counterpart normal skin (NS) and NS‐derived fibroblasts (NSFs). LPS could up‐regulate the expression of TLR4, pp65, Col I, Col III and α‐SMA in NSFs, but IL‐10 could down‐regulate their expression in both HSFs and LPS‐induced NSFs. Blocking IL‐10 receptor (IL‐10R) or the phosphorylation of STAT3, their expression was up‐regulated. In addition, *in vitro* and *in vivo* models results showed that IL‐10 could alleviate LPS‐induced fibroblast‐populated collagen lattice (FPCL) contraction and scar formation. Therefore, IL‐10 alleviates LPS‐induced skin scarring via IL‐10R/STAT3 axis regulating TLR4/NF‐κB pathway in dermal fibroblasts by reducing ECM proteins deposition and the conversion of fibroblasts to myofibroblasts. Our results indicate that IL‐10 can alleviate the LPS‐induced harmful effect on wound healing, reduce scar contracture, scar formation and skin fibrosis. Therefore, the down‐regulation of inflammation may lead to a suitable scar outcome and be a better option for improving scar quality.

## 1 INTRODUCTION

Skin injury activates physiological responses, which can be identified as inflammation, proliferation and remodelling phases.^1^, ^2^, ^3^ Although these phases are recognized as distinct entities, they usually overlap throughout the wound healing process. Inflammation may play a pivotal role in determining wound healing and scar outcome.^4^, ^5^, ^6^ The most desirable scar is thin, flat and almost invisible. Severe trauma (eg deep burns) and sometimes even standard surgery can result in adverse scar formation, known as a hypertrophic scar (HS).^2^, ^7^, ^8^

HS is a firm, raised, red, itchy, sometimes painful fibrotic skin disease that usually develops within 4‐8 weeks post‐injury and may recede over time.^2^, ^6^, ^7^, ^8^ Usually, the life quality of patients is declining because of loss of joint mobility and disfigurement.^6^, ^9^ There is currently no satisfactory clinical treatment for HS. The present studies suggest that HS is characterized by excessive abnormal deposition and metabolism disorders of collagen‐based extracellular matrix (ECM) proteins, mainly including type I collagen (Col I), type III collagen (Col III) and the transformation of fibroblasts to myofibroblasts during scar formation.^9^, ^10^, ^11^, ^12^, ^13^, ^14^ To date, the pathogenesis of HS has not been fully understood, but the role of bacterial contamination and prolonged wound inflammation is considered important.^2^, ^15^, ^16^, ^17^

IL‐10 is a potent anti‐inflammatory cytokine^18^, ^19^ that prevents fibrosis in several models, including dermal wounds,^20^, ^21^, ^22^, ^23^ myocardial infarction,^24^ lung^25^ and kidney injury.^26^ Emerging reports have shown that IL‐10 plays a key role in wound healing,^21^, ^23^, ^27^ and it has been identified as a promising therapeutic agent that can reduce scar formation.^12^, ^14^, ^23^, ^28^, ^29^, ^30^ However, the molecular mechanism of IL‐10‐mediated scar‐improvement is still unclear. IL‐10 can inhibit pro‐inflammatory mediators including TNF‐α, IL‐1β and IL‐8 and is thought to act through STAT3‐mediated signalling pathways.^21^, ^27^ Specifically, dimerized IL‐10s bind to an IL‐10 receptor (IL‐10R) complex. STAT3 is phosphorylated, inducing its dimerization and translocation into the nucleus to activate target gene expression.^21^, ^31^, ^32^ So far, whether the IL‐10R‐STAT3 pathway plays a major role in mediating the function of IL‐10 is still controversial.

Fibroblasts are one of the most important effector cell types responsible for scar formation.^12^, ^29^, ^30^ For a long time, HS fibroblasts (HSFs) have been considered to be responsible only for the overproduction of ECM components and the transformation of fibroblasts into myofibroblasts,^9^, ^10^, ^12^, ^13^, ^14^, ^30^ which is a major difference in comparison with normal skin fibroblasts (NSFs). Emerging evidence also shows that fibroblasts can also participate in immunological responses in direct response to pro‐inflammatory signals, as well as regulation of normal barrier function of the epithelium,^33^ infected tissue remodelling^34^, ^35^ and the infiltration behaviour of leucocytes to inflammatory sites.^36^, ^37^ Lipopolysaccharide (LPS) can activate toll‐like receptor 4 (TLR4) in dermal fibroblasts through NF‐κB, leading to the production of pro‐inflammatory cytokines, which in turn causes inflammation.^16^, ^38^

Based on the known anti‐inflammatory properties of IL‐10 and the lack of inflammation and inflammatory mediators in scarless wound repair, we suggested that modulation of the inflammatory response by IL‐10 would convert the wound healing phenotype from scar formation to scarless healing. To test this hypothesis, we evaluated the effect of IL‐10 on the production of inflammatory mediators and the presence of an inflammatory response in LPS‐stimulated NSFs, in an attempt to further clarify the role of IL‐10 during scar formation. Therefore, the aim of this study was to investigate whether IL‐10 can improve LPS‐induced inflammatory responses in dermal fibroblasts and skin scarring and to explore the possible mechanism of regulating TLR4/NF‐κB pathway during scar formation.

## 2 MATERIALS AND METHODS

### 2.1 Patients and tissue biopsy samples

Seven cases of HS and counterpart normal dermal skin (NS) tissues were collected from patients undergoing skin flap and skin graft plastic surgery at Xijing Hospital. In this study, the criteria for HSs collecting were ① no regression within 1 year and no medication before sampling; ② firm, raised (≥2 mm), red, inelastic and itchy in clinical diagnosis; and ③ identified by morphological and molecular biology methods in our laboratory (Figures S1‐S3). The information about patients was shown in Table 1. The written consent of all participants was obtained before the operation. All protocols used in this study have been approved by Ethics Committee of Xijing Hospital, affiliated to Fourth Military Medical University of China. HS and HSFs were used to compare with their counterpart NS and NSFs from the same patient.

**TABLE 1:** The profile of each sample

No. of patients	Duration (month)	Sex	Age (year)	HS site	NS site
C10423	6	Male	13	Neck	Abdomen
D60606	8	Female	27	Chest	Arm
D92524	9	Female	24	Shoulder	Arm
D54231	7	Male	36	Buttock	Abdomen
D60568	10	Male	12	Chest	Arm
E36211	5	Male	12	Shoulder	Arm
D54234	8	Female	43	Back	Back

### 2.2 Cell culture and treatment

Cell culture was performed as previously described.^23^, ^39^, ^40^ Briefly, fibroblasts were extracted from minced HS and their counterparts NS by incubation in a solution of type I collagenase (0.1 mg/mL; Sigma) at 37°C for 2.5 hours. The extracted HSFs and NSFs were collected and cultured at 37°C (in a 5% (v/v) CO_2_‐humidified incubator) in Dulbecco's modified Eagle's medium (DMEM; Gibco) supplemented with 10% foetal calf serum (FCS; Gibco), 100 U/mL penicillin and 100 U/mL streptomycin (Hyclone). All experiments were performed on the cells at passage 3‐5. The cultured HSFs and NSFs were identified by RT‐qPCR analysis (Figure S3).

### 2.3 Immunostaining and ultrastructure

Immunohistochemistry was performed as previously reported.^23^, ^39^, ^40^ In brief, the skin tissue fixed in 10% formalin buffer was embedded in paraffin blocks and cut into 4 μm‐thick tissue sections. The processed tissue sections were then dewaxed and treated with 3% H_2_O_2_ for 15 minutes, followed by blocked with goat serum for 30 minutes, incubated at 4°C overnight with a primary antibody against TLR4 (ab13867, 1:300, Abcam), p65 (ab32536, 1:200, Abcam) and immunostained with a SP‐9000 HistostainTM Kit (SP‐9000D, ZSGB), according to the manufacturer's instructions.

For immunofluorescence analysis,^23^, ^39^ cells were fixed in 4% formaldehyde for 30 minutes, washed with phosphate‐buffered saline (PBS), permeabilized with 0.1% Triton‐X100 for 10 minutes at room temperature, blocked with 1% bovine serum albumin (BSA), hybridized with a antibody specific for TLR4 (ab13867, 1:300, Abcam), p65 (ab32536, 1:200, Abcam) and pp65 (ab86299, 1:200, Abcam) at room temperature for 1 hours and then incubated with a Cy3‐conjugated goat secondary antibody (cw0159, 1:100, Cwbio, China) at 37°C for 1 hours. Finally, the samples were stained with 4′,6′‐diamidino‐2‐phenylindole (DAPI, Sigma).

### 2.4 RT‐qPCR

RT‐qPCR was performed as previously reported.^23^, ^39^, ^40^ In brief, the total RNA was extracted from cultured cells using an RNA isolation kit (Takara, Japan). The purity of RNA was calculated as follows: A260/A280 (1.9‐2.0). Table 2 lists the human primer pairs used to amplify genes from cDNA templates. The mRNA levels of genes were normalized to the housekeeping gene encoding GAPDH.

**TABLE 2:** Sequences of primers for RT‐qPCR

Gene	Forward primer (5′→3′)	Reverse primer (5′→3′)
Col I	gagggcaacagcaggttcactta	tcagcaccaccgatgtcca
Col III	ccacggaaacactggtggac	gccagctgcacatcaaggac
α‐SMA	gccaatggctctgggctctgtaa	tgtgcttcgtcacccacgta
TLR4	ggccattgctgccaacat	caacaatcacctttcggctttt
p65	tgctgtgcggctctgcttcc	aggctggggtctgcgtaggg
GAPDH	gcaccgtcaagctgagaac	tggtgaagacgccagtgaa

### 2.5 Western blot

Cultured fibroblasts, with a 70%‐80% confluence after incubation for 12‐16 hours in serum‐free medium, were stimulated with LPS (l‐2880, 1.0 μg/mL, Sigma), IL‐10 (#200‐10, 10 ng/mL, PeproTech), IL10RA (sc‐365374, 1:500, Santa Cruz) or cryptotanshinone (s2285, 4.6 μmol/L, Selleckchem) for 48 hours. As for the inhibitory role of IL‐10 signalling, siRNAs for IL10Rɑ (NM001558.3) were also used, siIL10Rɑ sense: 5′‐gucugaaaguaccugcuauga‐3′, anti‐sence: 5′‐ucauagcagguacuuucagac‐3′. Using 50 nmol/L siRNA fragment, the transfection incubation time for siRNA/Lipofectamine RNAiMAX reagent complexes was 48 hours, then washed in PBS and resuspended in RIPA cell lysis solution (Beyotime) supplemented with 200 μg/mL phenylmethylsulfonyl fluoride (PMSF, Boster), phosphatase inhibitor cocktail (Sigma) and protease inhibitor cocktail (Sigma). The BCA assay (Pierce) was used to determine the protein concentration of the cell lysate. Then, the Western blotting was performed as previously described.^23^, ^39^, ^40^

### 2.6 Inflammatory cytokines assay

Inflammatory cytokines produced by cultured fibroblasts and determined using QAH‐INF‐1 array (Raybiotech) according to the manufacture's instruction by Wayen Biotechnologies Inc. Fibroblasts were grown to 70% confluence, and then, the media were changed to FBS‐free DMEM for 12‐16 hours. The cells were then stimulated with 1.0 mg/mL of LPS (Sigma) and cultured for 48 hours. After final culture, the media were collected for analysis of inflammatory cytokines.

### 2.7 FPCL contractility and improvement assays

Fibroblast‐populated collagen lattice (FPCL) contractility assays proceeded as previously described.^23^, ^41^, ^42^ Briefly, collagen lattices were polymerized in 24‐well tissue culture plate (Corning). 3.15 mL of rat tail tendon collagen (1.2 mg/mL) was mixed with 0.9 mL 5×DMEM in a 10 mL centrifuge tube cooled on ice. The pH was adjusted to a range of 7.2‐7.5. Trypsinize the cells from the confluent tissue culture flask, and 0.45 mL of the cell suspension (containing 9 × 10^5^ NSFs) was added to the collagen solution, gently mixed and added into the 24‐well plate (500 μL per well). Collagen lattices were allowed to gel at 37℃for 25 minutes in a 5% (v/v) CO_2_ humidified atmosphere. Incubate the gel for 24 hours, then separate it from the surface of the wells by marginalizing the lattice with a sterile spatula and gently rotating the 24‐well plate. After 48 hours of detachment from the surface of the well, FPCL was analysed by optical microscopy.

### 2.8 Animal model and treatment

A rabbit ear scar model was according to previous description.^39^, ^43^, ^44^ Male New Zealand white rabbits weighing 2.0‐2.5 kg were purchased from Experimental Animal Center of Fourth Military Medical University and approved by Experimental Animal Committee of Fourth Military Medical University. Animal experiments were carried out in our laboratory at Xijing hospital, affiliated to Fourth Military Medical University of China. The rabbits were housed in separate cages and reared under standard conditions at RT (22‐24℃) in a 12‐h light/12‐h dark cycle. They were anaesthetized by intravenous administration of sodium pentobarbital (30 mg/kg). In a sterile environment, four round wounds with a diameter of 10 mm down to the cartilage on each ear were randomly created on each ear. On the 28th day after surgery, the scars randomly placed into PBS, LPS and IL‐10+ LPS treatment groups (6 scars for each group). IL‐10 was injected into the scars for 24 hours before LPS injection, and they were applied to scars two times in a week.

### 2.9 Statistical analysis

All results were obtained from at least three independent experiments and analysed using SPSS 20.0 software as the mean ± standard error (SEM). Quantitative data between two groups were analysed using Student's t test, and the comparisons among multiple groups were conducted using analysis of variance followed by Turkey's post hoc test. A value of *P* < .05 was considered statistically significant.

## 3 RESULTS

### 3.1 HS is a serious fibrotic skin disease characterized by excessive ECM proteins deposition

HE staining showed an abundance of fibroblasts in HS (Figure S1A,C), whereas a lower density of fibroblasts was observed in their counterparts NS (Figure S1B,C, *P ˂ .05). Masson staining showed the excessive collagen deposition in HS (Figure S1D,F), whereas less deposition was observed in their counterparts NS (Figure S1E,F, ***P ˂ .001). In order to clarify the different expression of fibrotic proteins between HS and NS, Col I, Col III and α‐SMA were analysed by immunohistochemistry. The results showed that the relative density of Col I (Figure S2A‐C, ***P ˂ .001), Col III (Figure S2D‐F, *P ˂ .05) and α‐SMA (Figure S2G‐I, **P ˂ .01) was higher in HSs than their counterparts NSs. RT‐qPCR results showed that the transcription levels of Col I, Col III and α‐SMA were significantly higher in HS/HSFs than the counterparts NS/NSFs (Figure S3, *P ˂ 0.05, **P ˂ 0.01). These results show that HS is a significant fibrotic skin disease characterized by a dysregulation of collagen‐based ECM proteins deposition. The results are similar to the previous works,^9^, ^10^, ^12^, ^13^, ^14^, ^30^ confirming the reliability of the samples.

### 3.2 Key inflammatory molecules in TLR4/NF‐κB pathway are elevated in HS and HSFs

**FIGURE 1:**
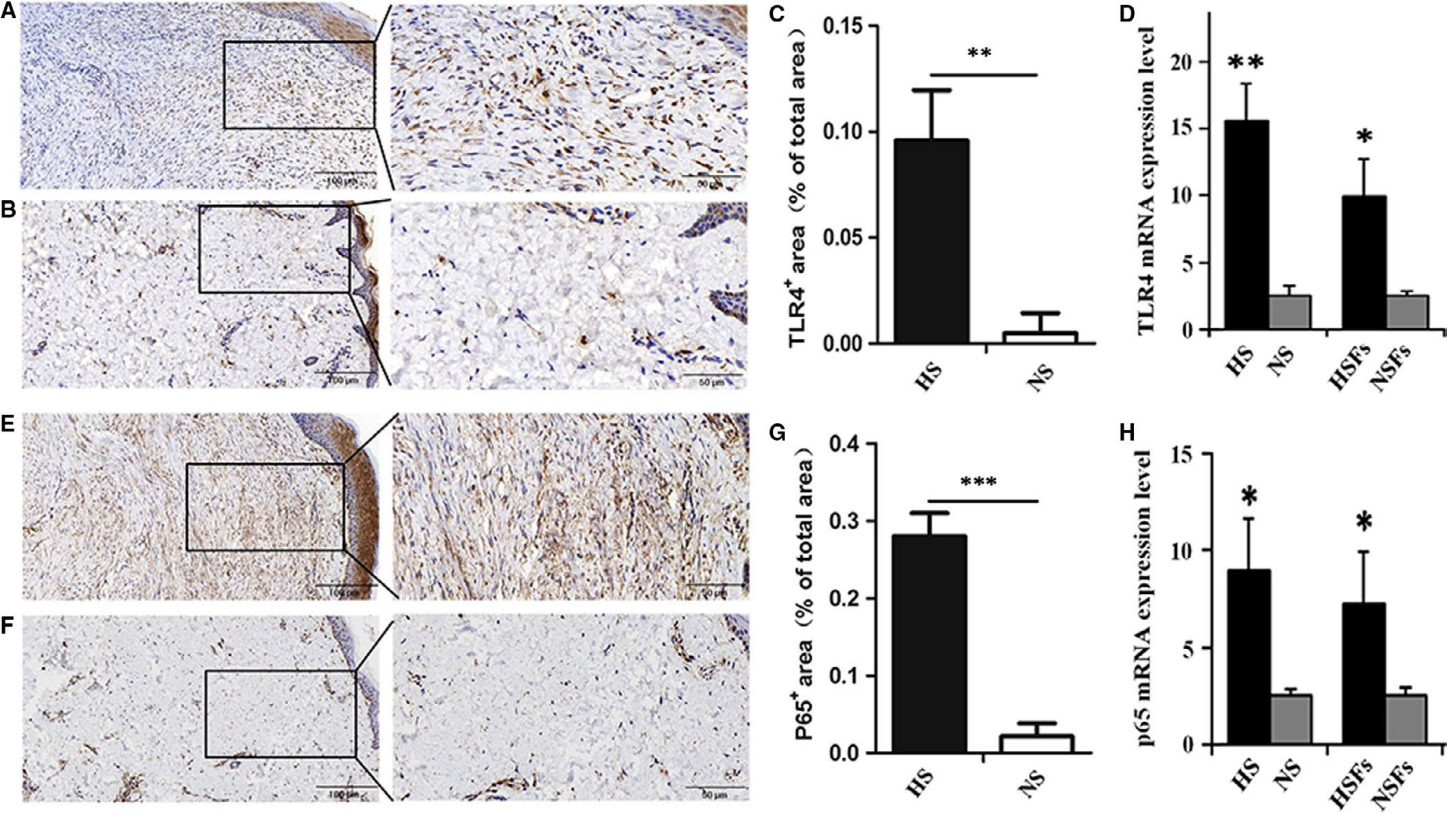
Key inflammatory molecules in TLR4/NF‐κB pathway are elevated in HS and HSFs. A, LR4 was detected by streptavidin‐peroxidase DAB staining in HS. B, TLR4 was detected by streptavidin‐peroxidase DAB staining in NS. C, Ratio of TLR4 positive cells in HS and its counterpart NS. D, The mRNA level of TLR4 was analysed by RT‐qPCR in HS/HSFs and their counterparts NS/NSFs. E, pp65 was detected by streptavidin‐peroxidase DAB staining in HS. F, pp65 was detected by streptavidin‐peroxidase DAB staining in NS. G, Ratio of pp65 positive area in HS and its counterpart NS. H, The mRNA level of p65 was analysed by RT‐qPCR in HS/NS, and their counterparts HSFs/NSFs (A, B, E, F, Scale bars, 100 μm, 50 μm; Data are expressed as the mean ± SEM, n = 6); (D, H, n = 3; *P < .05, **P < .01, ***P < .001 compared with the counterpart NS/NSFs)

Immunohistochemistry was performed to evaluate TLR4 and pp65 in HS and their counterparts NS. The result showed that the positively stained fibroblasts of TLR4 (Figure 1A‐C) and pp65 (Figure 1E‐G) were remarkably up‐regulated to 85% (Figure 1A‐C, **P ˂ .01) and 87% (Figure 1E‐G, ***P ˂ .001) in HS than NS, respectively. RT‐qPCR result showed the mRNA levels of TLR4 and p65 in HS/HSFs were much higher compared to NS/NSFs (Figure 1D,H, *P ˂ .05, **P ˂ .01). Immunofluorescence results showed that TLR4, pp65 and p65 were also expressed in cultured HSFs (Figure S4). These findings confirm that TLR4 and pp65 are existed in HS/HSFs and higher expressed in HS/HSFs than their counterparts NS/NSFs.

### 3.3 LPS induces NSFs to HSFs and participates in HS formation

**FIGURE 2:**
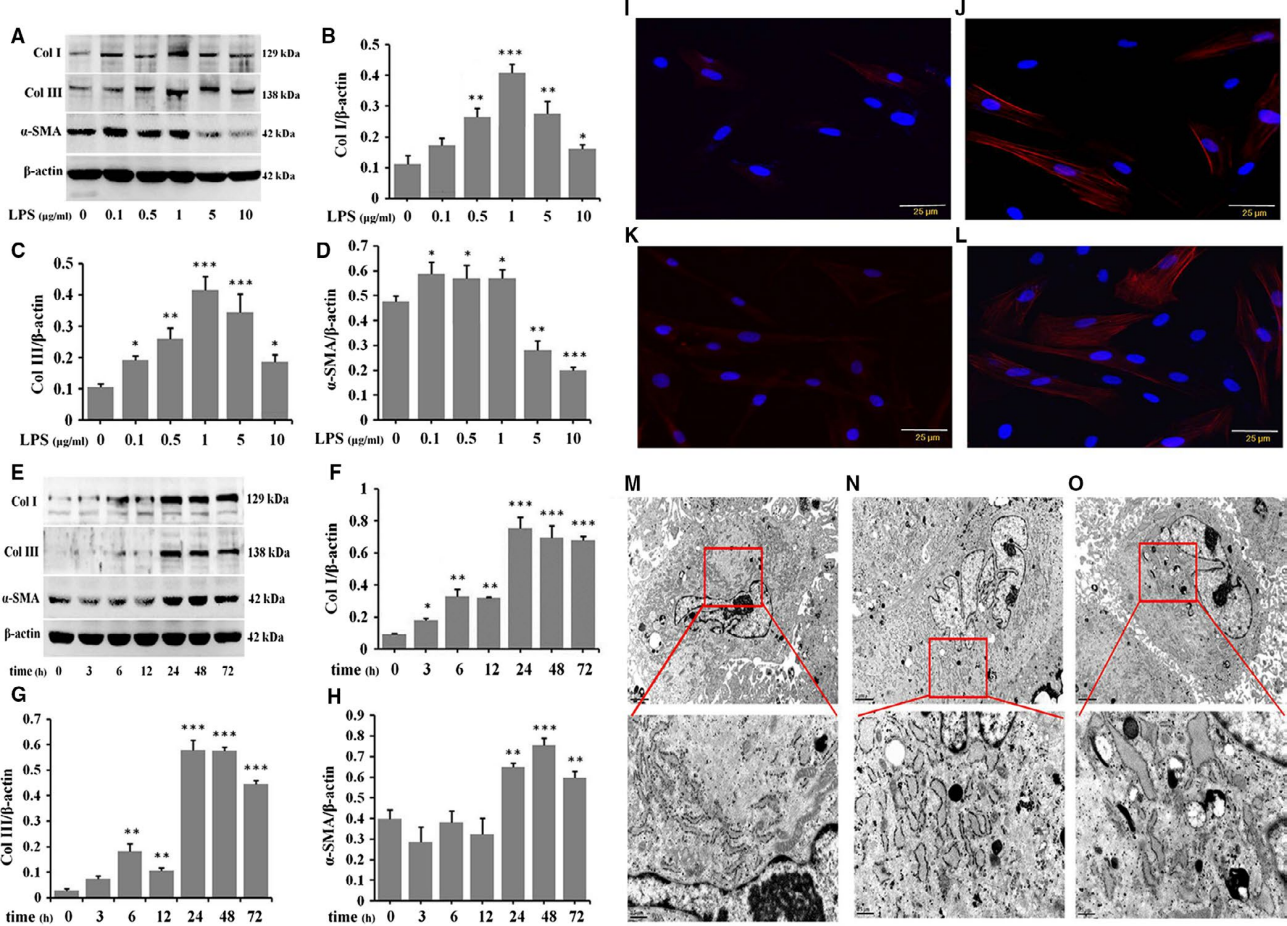
LPS induces the inversion of NSFs to HSFs and participates in HS formation. NSFs were stimulated with different dose of LPS for 48 h, A, Western blot result of Col I, Col III and α‐SMA. B, Ratio of Col I to β‐actin. C, Ratio of Col III to β‐actin. D, Ratio of α‐SMA to β‐actin. NSFs were stimulated with 1.0 μg/mL LPS at different time‐points, E, Western blot result of Col I, Col III and α‐SMA. F, Ratio of Col I to β‐actin. G, Ratio of Col III to β‐actin. H, Ratio of α‐SMA to β‐actin, A‐H, Data are expressed as the mean ± SEM; n = 3; *P < .05, **P < .01, ***P < .001 compared with control. I, Immunofluorescence for α‐SMA in NSFs. J, Immunofluorescence for α‐SMA in LPS‐stimulated NSFs. K, Immunofluorescence for α‐SMA in HSFs. L, Immunofluorescence for α‐SMA in LPS‐stimulated HSFs, I‐L, Scale bars, 25 μm. M, Ultrastructure of NSFs. N, Ultrastructure of HSFs. O, Ultrastructure of LPS‐stimulated NSFs. The ultrastrcture parameter was evaluated at least 6 fibroblasts in each sample. M‐O, Scale bars, 2 μm, 0.5 μm

To investigate whether the observed effects after LPS treatment were owing to its impact on ECM proteins expression in cultured NSFs. Stimulated NSFs with 0, 0.1, 0.5, 1.0, 5.0 and 10 μg/mL LPS and cultured 48 hours, Western blot result showed Col I and Col III significant expression in 0.5, 1.0 and 5 μg/mL LPS‐stimulated groups (Figure 2A‐C, *P ˂ .05, **P ˂ .01, ***P ˂ .001), and α‐SMA significant expression in 0.1, 0.5 and 1.0 μg/mL LPS‐stimulated groups than those at the other concentrations LPS‐stimulated groups (Figure 2A,D, *P ˂ .05). To determine the LPS‐induced fibrotic proteins, we tested the expression of Col I, Col III and α‐SMA at 0‐72 hours after 1.0 μg/mL LPS‐stimulated NSFs, Western blot result showed that Col I, Col III and α‐SMA progressively increased from 3 to 72 hours after LPS treatment compared with control group, and the peak expression levels were at 24‐48 hours (Figure 2E‐H, *P ˂ .05, **P ˂ .01, ***P ˂ .001). Therefore, in LPS‐stimulated NSF, the peak expression of fibrotic protein was at 48 hours.

During the progression of HS, fibroblasts are activated and transformed into myofibroblast, which can be identified by the expression of α‐SMA, and myofibroblast secretes abundant levels of collagen‐based ECM proteins that cause scar formation.^6^, ^7^, ^10^, ^12^, ^13^, ^14^, ^30^ To further validate whether LPS could convert NSFs to HSFs and participate in HS formation, NSFs were treated with 1.0 μg/mL LPS for 48 hours, and the immunofluorescence result showed that α‐SMA positively stained fibroblasts were higher in HSFs (Figure 2K), LPS‐stimulated NSFs (Figure 2J) and LPS‐stimulated HSFs (Figure 2L) than in NSFs (Figure 2I).

The ultrastructural morphology of NSFs, LPS‐stimulated NSFs, and HSFs was compared by transmission electron microscope (TEM, Figure 2M‐O). Treatment of NSFs with 1.0 μg/mL LPS resulted in diversified morphological change in the ultrastructure of fibroblasts, resulting in high levels of cellular organelles, such as endoplasmic reticulum, mitochondria, lysosome, vesicular structure and autolysosome (Figure 2M‐O). Notably, the ultrastructure of HSFs mimicked the ultrastructure of LPS‐stimulated NSFs (Figure 2N,O). These results suggest that LPS can convert NSFs to HSFs and regulate HS formation. Taken together, LPS plays an important role during scar formation and skin fibrosis.

### 3.4 LPS induces the expression of key molecules in TLR4/NF‐κB pathway and the downstream inflammatory cytokines in NSFs

**FIGURE 3:**
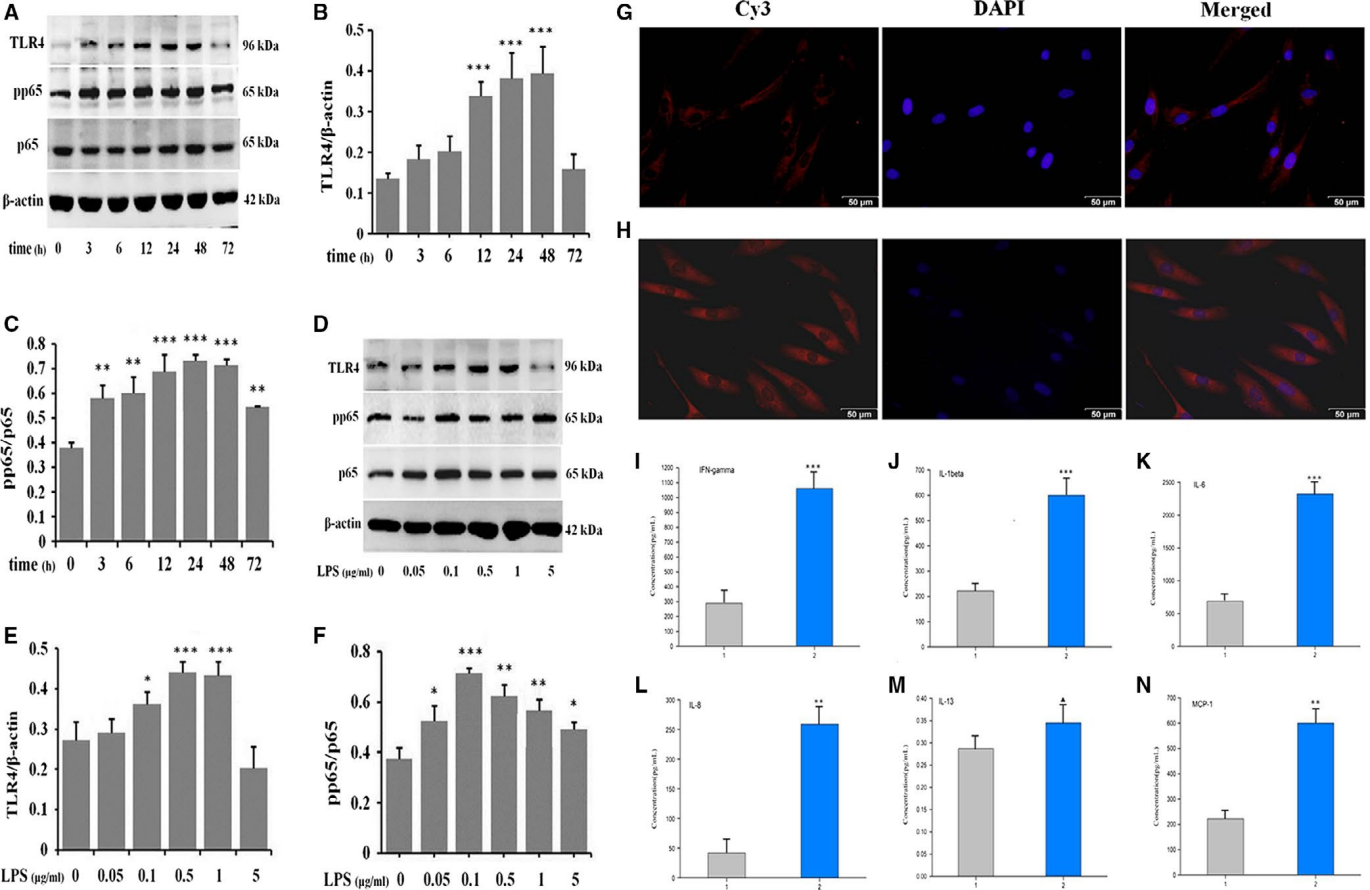
LPS induces the expression of key molecules in TLR4/NF‐κB pathway and the production of inflammatory cytokines in NSFs. NSFs were stimulated with 1.0 μg/mL LPS at different time‐points, A, Western blot result of TLR4 and pp65. B, Ratio of TLR4 to β‐actin. C, Ratio of pp65 to p65. NSFs were stimulated with different dose of LPS for 48 h. D, Western blot result of TLR4 and pp65. E, Ratio of TLR4 to β‐actin. F, Ratio of pp65 to p65. Data are expressed as the mean ± SEM; n = 3; *P < .05, **P < .01, ***P < .001 compared with control. G, Immunofluorescence for TLR4 in NSFs. H, Immunofluorescence for TLR4 in LPS‐stimulated NSFs. g, h, Scale bars, 50 μm. Inflammatory cytokines array result in LPS‐stimulated NSFs, I, IFN‐γ. J, IL‐1β. K, IL‐6. L, IL‐8. M, IL‐13. N, MCP‐1. (1. the supernatant of NSFs; 2. the supernatant of LPS‐stimulated NSFs). Data are expressed as the mean ± SEM; n = 3; ^▲^ *P* > .05, **P < .01, ***P < .001 compared with controls)

The next to investigate was whether the observed effects after LPS treatment were owing to its impact on the key molecules in TLR4/NF‐κB pathway and the downstream inflammatory cytokines. After treating NSFs with 1.0 μg/mL LPS, Western blot results showed that TLR4 and pp65 progressively increased from 3 to 72 hours compared to control group (Figure 3A‐C, **P ˂ .01, ***P ˂ .001), and the peak expression of TLR4 was from 12 to 48 hours (Figure 3A,B), and pp65 was from 3 to 72 hours (Figure 3A,C). The expression of TLR4 and pp65 was decreased in LPS‐stimulated NSFs at 72 hours (Figure 3A‐C).

Afterwards, NSFs were stimulated with 0, 0.05, 0.1, 0.5, 1.0 and 5.0 μg/mL LPS for 48 hours. Western blot results showed significant TLR4 expression in 0.1, 0.5 and 1.0 μg/mL LPS‐stimulated groups (Figure 3D,E, *P ˂ .05, ***P ˂ .001), and significant pp65 expression in 0.1, 0.5, 1.0 and 5 μg/mL LPS‐stimulated groups (Figure 3D,F, *P ˂ .05, **P ˂ .01, ***P ˂ .001). Immunofluorescence results showed that TLR4 was higher expressed in LPS‐stimulated NSFs group (Figure 3H) than in NSFs group (Figure 3G). These results suggest that LPS may promote the transition from NSFs to HSFs in which the TLR4 is higher expressed.

Next, we analysed the effect of LPS on inflammatory cytokines in cultured NSFs. QAH‐INF‐1 array result showed that the production of IFN‐γ (Figure 3I), IL‐1β (Figure 3J), IL‐6 (Figure 3K), IL‐8 (Figure 3L) and MCP‐1 (Figure 3N) in LPS‐stimulated NSFs group were increased about 3‐5 times compared to NSFs group (Figure 3I‐N, **P ˂ .01, ***P ˂ .001).

Our results suggest that LPS induces NSFs to up‐regulate TLR4, pp65 and the downstream inflammatory cytokines, such as IFN‐γ, IL‐1β, IL‐6, IL‐8, IL‐13 and MCP‐1. This means LPS can induce NSFs to product inflammatory cytokines. Therefore, these results provide insight on the importance of inflammatory regulation in scar formation and skin fibrosis.

### 3.5 IL‐10 suppresses the expression of inflammatory molecules and ECM proteins in HSFs and LPS‐stimulated NSFs

**FIGURE 4:**
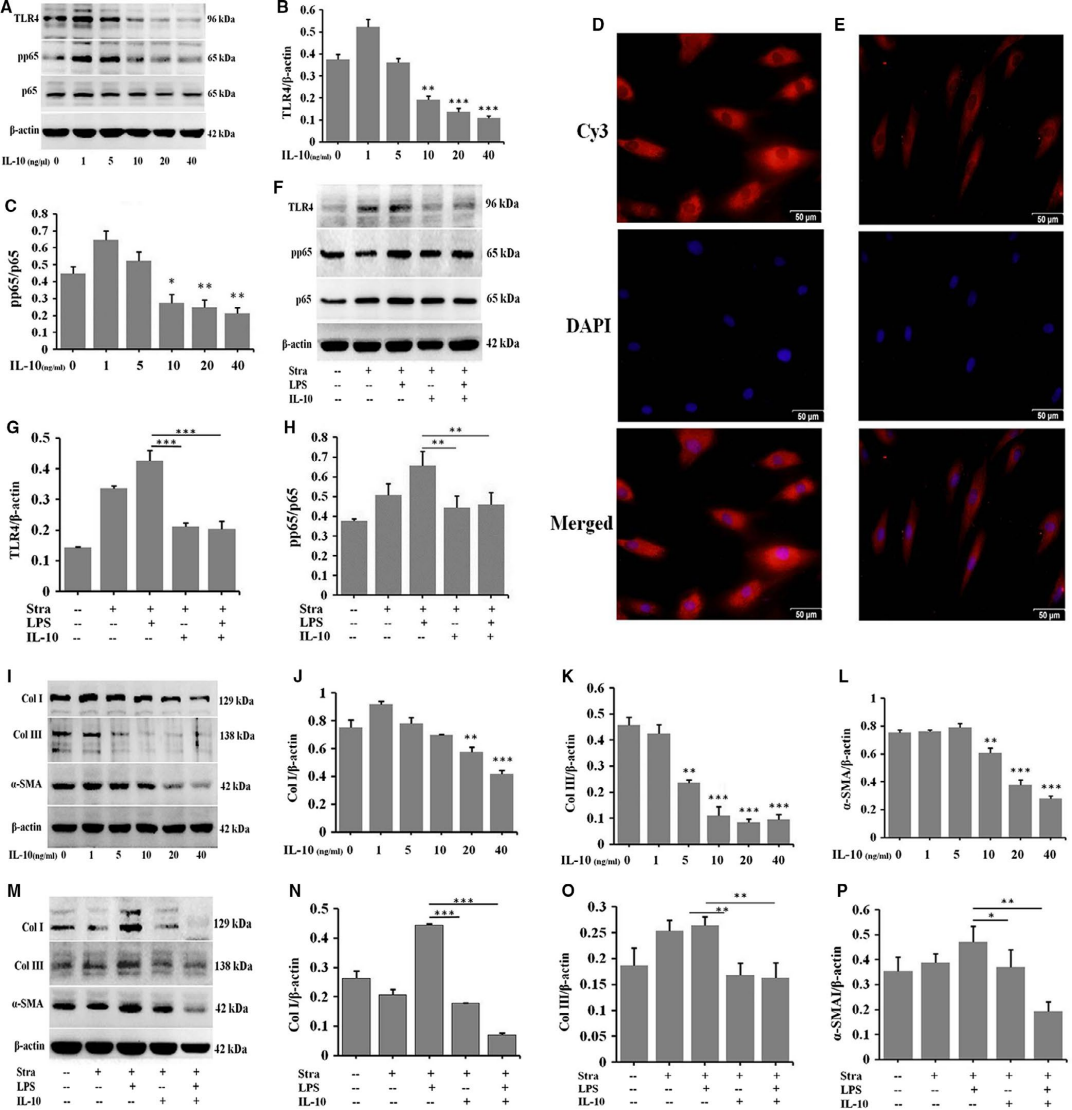
IL‐10 suppresses the expression of inflammatory molecules and ECM proteins in HSFs and LPS‐stimulated NSFs. HSFs were stimulated with different dose of IL‐10 for 48 h, A, Western blot result of TLR4 and p65. B, Ratio of TLR4 to β‐actin. C, Ratio of pp65 to p65. D, Immunofluorescence for TLR4 in HSFs. E, Immunofluorescence for TLR4 in IL‐10‐stimulated HSFs (D, E, Scale bars, 50 μm). NSFs with 70%‐80% confluent were starved by culturing in serum‐depleted medium for 12‐16 h before exposure to IL‐10 and LPS for 48 h, F, Western blot result of TLR4 and pp65. G, Ratio of TLR4 to β‐actin. H, Ratio of pp65 to p65. HSFs were stimulated with different dose of IL‐10, I, Western blot result of Col I, Col III and α‐SMA. J, Ratio of Col I to β‐actin. K, Ratio of Col III to β‐actin. L, Ratio of α‐SMA to β‐actin. NSFs were stimulated with IL‐10 and LPS, M, Western blot result of Col I, Col III and α‐SMA. N, Ratio of Col I to β‐actin. O, Ratio of Col III to β‐actin. P, Ratio of α‐SMA to β‐actin. Data are expressed as the mean ± SEM; n = 3, *P ˂ .05, **P ˂ .01, ***P ˂ .001 compared with controls

IL‐10 is a potent anti‐inflammatory cytokine^18^, ^19^ and plays a pivotal role in wound healing.^21^, ^23^, ^27^ Recently, it has been identified as a promising new therapeutic agent that can reduce scar formation.^12^, ^14^, ^23^, ^28^, ^29^, ^30^ After treatment of HSFs with 0, 1, 5, 10, 20 and 40 ng/mL IL‐10 for 48 hours, Western blot results showed that TLR4 and pp65 were significantly suppressed in 10, 20 and 40 ng/mL groups compared to control group (Figure 4A‐C, *P ˂ .05, **P ˂ .01, ***P ˂ .001). Immunofluorescence results showed that TLR4 was lower expressed in 20 ng/mL IL‐10‐stimulated HSFs (Figure 4E) than in HSFs (Figure 4D). To further investigate the effect of IL‐10 on inflammatory molecules in LPS‐stimulated NSFs, Western blot results showed that the expression of TLR4 and pp65 were increased in LPS‐stimulated NSFs and decreased in IL‐10‐stimulated NSFs (Figure 4F‐H, **P ˂ .01, ***P ˂ .001), whereas adding LPS to IL‐10‐stimulated NSFs, they were significantly decreased (Figure 4F‐H, **P ˂ .01, ***P ˂ .001). These results indicate that IL‐10 suppresses the production of inflammatory molecules in LPS‐stimulated NSFs.

After treatment of HSFs with 1, 5, 10, 20 and 40 ng/mL IL‐10 for 48 hours, Western blot results showed that the expression of Col I, Col III and α‐SMA was progressively down‐regulated compared to HSFs group (Figure 4I‐L, **P ˂ .01, ***P ˂ .001). To investigate the effect of IL‐10 on fibrotic proteins in LPS‐stimulated NSFs, the protein levels of Col I, Col III and α‐SMA were detected by Western blot. The results showed that the proteins were significantly increased in LPS‐stimulated NSFs and decreased in IL‐10‐stimulated NSFs, whereas adding LPS to IL‐10‐stimulated NSFs, they were also decreased (Figure 4M‐P, *P ˂ .05, **P ˂ .01, ***P ˂ .001).

These results suggest that IL‐10 inhibits the production of inflammatory molecules and the deposition of ECM proteins in LPS‐stimulated NSFs. Therefore, a down‐regulation of inflammation may lead to a suitable scar outcome.

### 3.6 IL‐10 regulates TLR4/NF‐κB signalling pathway through IL‐10R/STAT3 pathway

**FIGURE 5:**
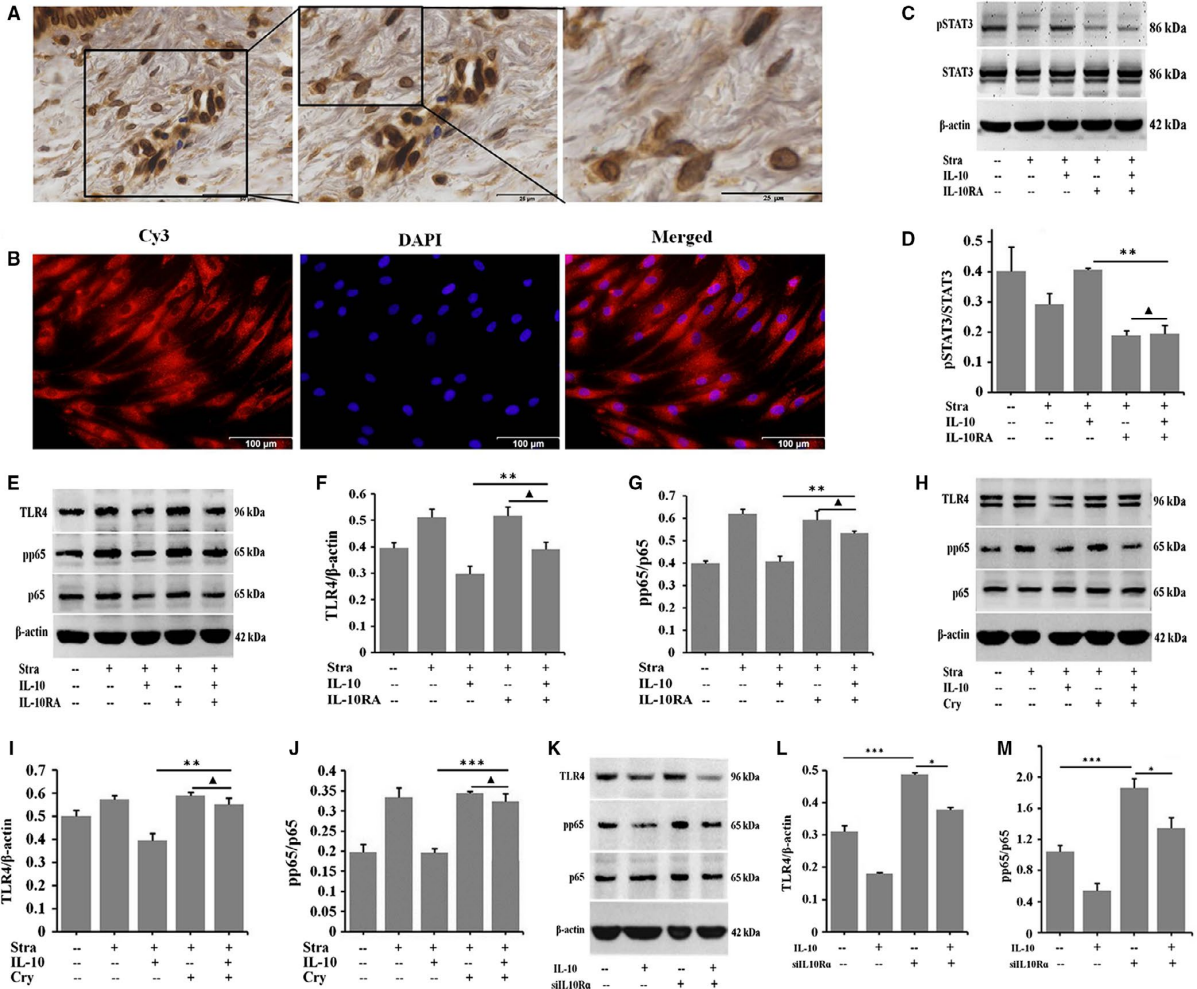
IL‐10 regulates TLR4/NF‐κB inflammatory signal by IL‐10R/STAT3 pathway. A, Immunohistochemistry for IL‐10R in NS tissues. Scale bars, 50 μm, 25 μm. B, Immunofluorescence for IL‐10R in NSFs. Scale bars, 100 μm. NSFs with 70%‐80% confluent were starved in serum‐depleted medium for 12‐16 h before exposure to IL‐10 and IL‐10RA for 30 min, C, Western blot result of pSTAT3 and STAT3. D, Ratio of pSTAT3 to STAT3. HSFs with 70%‐80% confluent were starved in serum‐depleted medium for 12‐16 h before exposure to IL‐10, IL‐10RA/cryptotanshinone or transfecting siIL10Rɑ for 48 h, E, Western blot result of TLR4 and pp65 in HSFs. F, Ratio of TLR4 to β‐actin. G, Ratio of pp65 to p65. H, Western blot result of TLR4 and pp65 in HSFs. I, Rate of TLR4 to β‐actin. J, Ratio of pp65 to p65. K, Western blot result of TLR4 and pp65 in HSFs. L, Rate of TLR4 to β‐actin. M, Ratio of pp65 to p65. Data are expressed as the mean ± SEM; n = 3, ^▲^ *P* > .05, *P ˂ 0.05, **P ˂ .01, ***P ˂ .001 compared with controls

IL‐10 is thought to function by its receptor (IL‐10R) via STAT3‐mediated (IL‐10R/STAT3) signalling pathway.^21^, ^27^ The immunostaining results showed that there were amount of IL‐10R‐positive fibroblasts in NS (Figure 5A) and NSFs (Figure 5B). After treatment of HSFs with 0, 1, 5, 10, 20 and 40 ng/mL IL‐10, Western blot results showed that the expression of pSTAT3 was up‐regulated in a dose‐dependent manner (Figure S5, *P ˂ .05, **P ˂ .01, ***P ˂ .001). To further verify the result, pSTAT3 in response to IL‐10 treatment was assessed in the presence or absence of a function‐blocking antibody against the IL‐10R (IL‐10RA). IL‐10RA could reduce pSTAT3 (Figure 5C,D, **P ˂ .01) in IL‐10‐stimulated HSFs. To confirm whether IL‐10 exerts its anti‐inflammatory action through the activation of IL‐10R/STAT3 signal transduction pathways in HSFs, IL‐10RA and cryptotanshinone were used to block IL‐10R and pSTAT3. As shown in Figure 5E‐J, IL‐10 significantly down‐regulated the expression of TLR4 and pp65. After IL‐10RA treatment of IL‐10‐stimulated NSFs, the expression of TLR4 and pp65 was significantly up‐regulated (Figure 5E‐G, **P ˂ .01). After blocking the phosphorylation of STAT3 by cryptotanshinone, the expression of pp65 was also up‐regulated (Figure 5H‐J, **P ˂ .01, ***P ˂ .001) in HSFs. In addition, siIL10Rɑ significantly up‐regulated the expression of TLR4 and pp65 in HSFs (Figure 5K‐M, ***P ˂ .001). After IL‐10 treatment, the expression of TLR4 and pp65 was down‐regulated in some extend (Figure 5K‐M, *P ˂ .05). Therefore, the anti‐inflammatory function of IL‐10 is through regulating TLR4 and pp65 in TLR4/NF‐κB pathway.

### 3.7 IL‐10 inhibits LPS‐induced the deposition of ECM proteins by IL‐10R/STAT3 axis regulating TLR4/NF‐κB pathway

**FIGURE 6:**
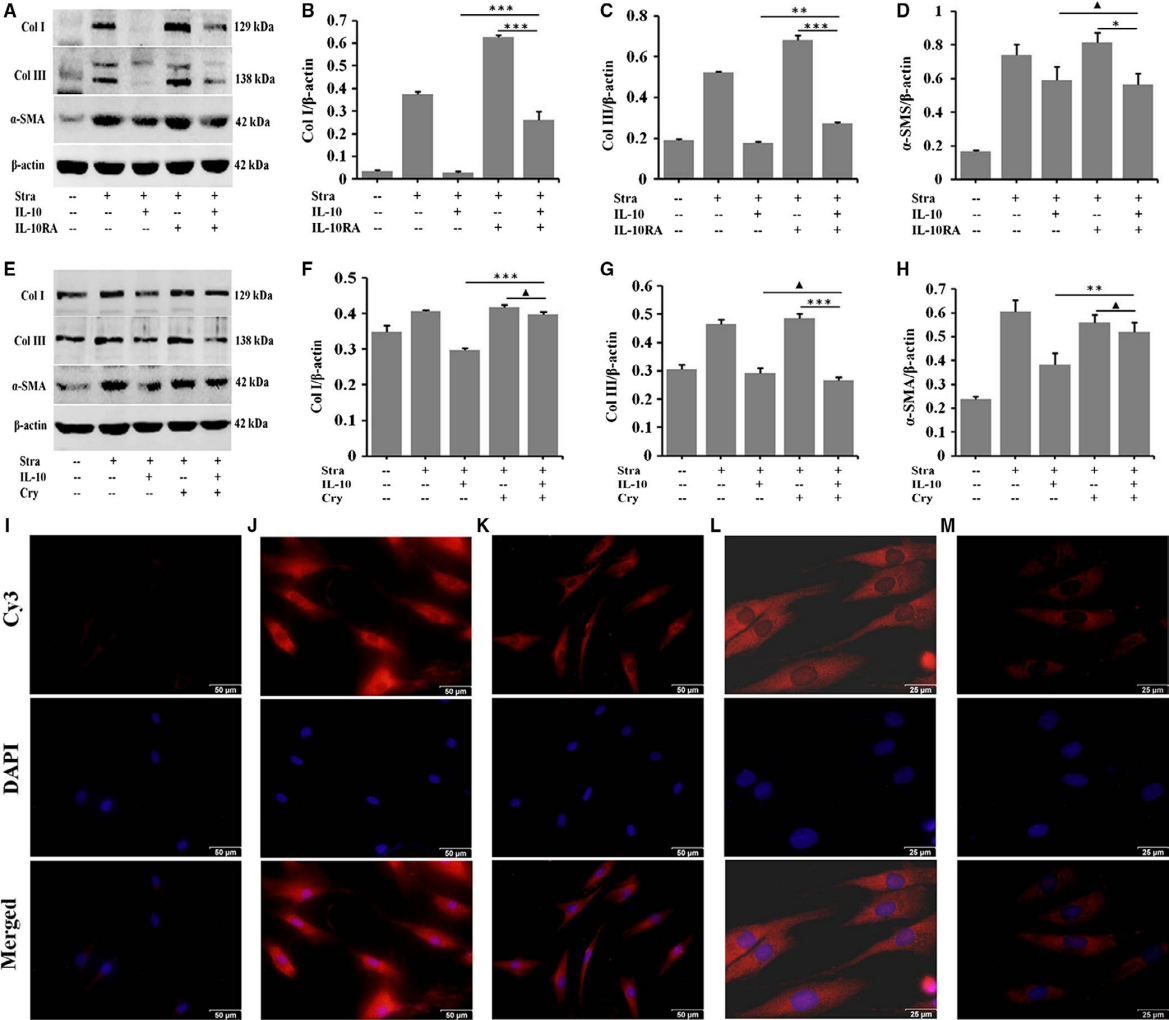
IL‐10 inhibits LPS‐induced deposition of ECM proteins by IL‐10R/STAT3 axis regulating TLR4/NF‐κB pathway. NSFs with 70%‐80% confluent were starved in serum‐depleted medium for 12‐16 h before exposure to IL‐10 and IL‐10RA or cryptotanshinone for 48 h, A, Western blot result of Col I, Col III and α‐SMA. B, Ratio of Col I to β‐actin. C, Ratio of Col III to β‐actin. D, Ratio of α‐SMA to β‐actin. E, Western blot result of Col I, Col III and α‐SMA. F, Ratio of Col I to β‐actin. G, Ratio of Col III to β‐actin. H, Ratio of α‐SMA to β‐actin. Data are expressed as the mean ± SEM; n = 3, ^▲^ *P* > .05, *P ˂ .05, **P ˂ .01, ***P ˂ .001 compared with controls. Immunofluorescence for Col I and α‐SMA in HSFs and IL‐10‐stimulated HSFs. I, The isotype control. J, Immunofluorescence for Col I in HSFs. K, Immunofluorescence for Col I in IL‐10‐stimulated HSFs. L, Immunofluorescence for α‐SMA in HSFs. M, Immunofluorescence for α‐SMA in IL‐10‐stimulated HSFs. I‐M, Scale bars, 50 μm, 25 μm

To confirm whether IL‐10 exerts its anti‐fibrotic action through the IL‐10R/STAT3 and TLR4/NF‐κB signalling pathways, IL‐10RA and cryptotanshinone (Cry) were, respectively, used to block IL‐10R and pSTAT3 in LPS‐stimulated NSFs for 48 hours. Western blot results showed that IL‐10 significantly down‐regulated the expression of Col I, Col III and α‐SMA. After IL‐10RA (Figure 6A‐D) and Cry (Figure 6E‐H) treatment, Col I, Col III and α‐SMA were significantly up‐regulated in IL‐10‐stimulated NSFs (Figure 6A‐H, *P ˂ .05, **P ˂ .01, ***P ˂ .001). More importantly, the immunostaining results confirmed that IL‐10 significantly down‐regulated the expression of Col I (Figure 6J,K) and α‐SMA (Figure 6L,M) in LPS‐stimulated NSFs. Therefore, IL‐10 inhibits LPS‐induced fibrosis by IL‐10R/STAT3 axis regulating TLR4/NF‐κB pathway.

### 3.8 IL‐10 inhibits LPS‐induced FPCL contracture

**FIGURE 7:**
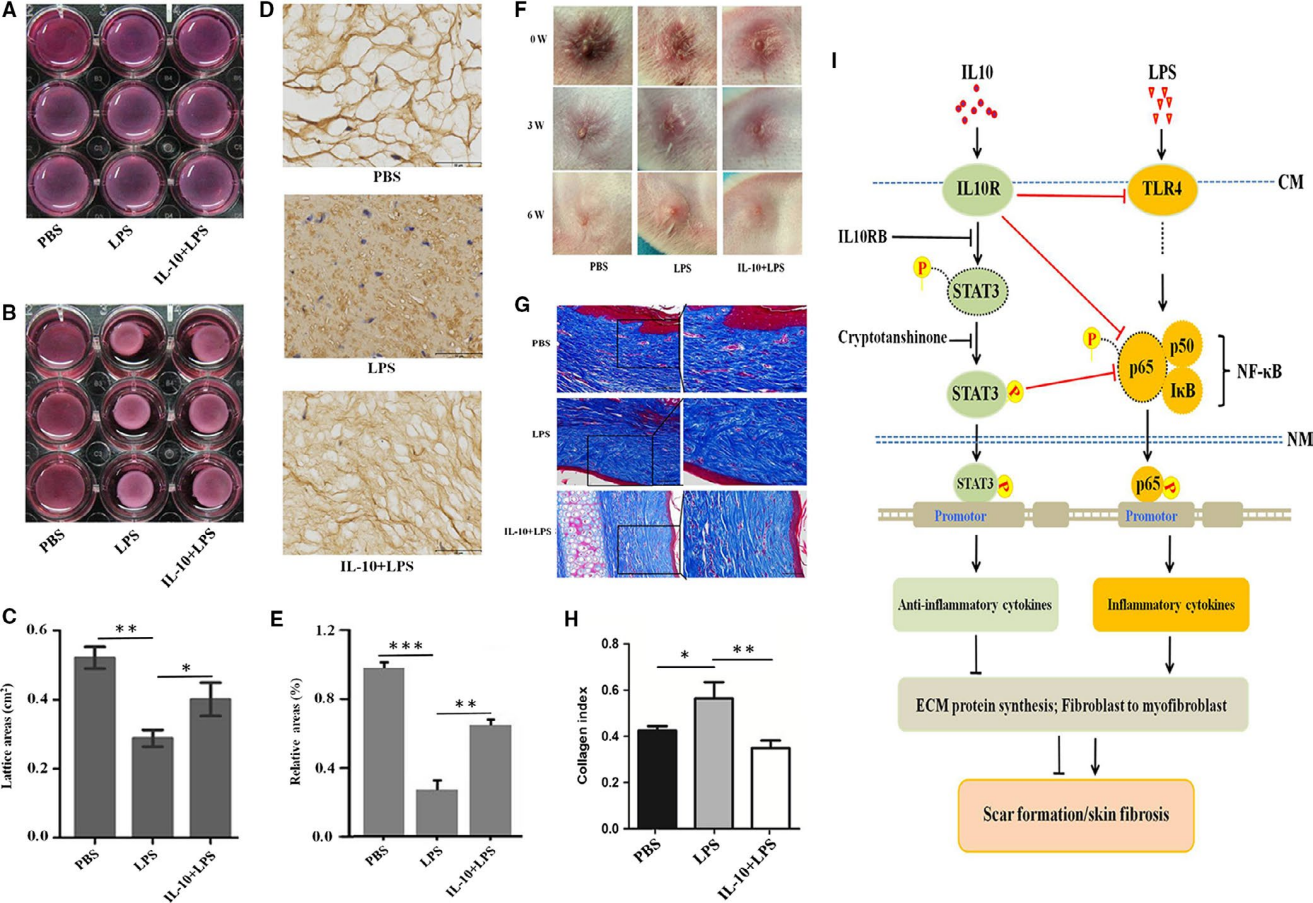
IL‐10 reduces LPS‐induced scar formation *in vivo* and *in vitro*. A, FPCLs were detached from the edge of the well. B, FPCLs treatment with PBS, LPS and IL‐10 + LPS for 48 h. C, FPCL surface area was determined to quantify contraction at 48 h. FPCLs were fixed in 4% paraformaldehyde, sectioned and stained with immunohistochemistry for Col I, D, Reticular structure of FPCLs in PBS, LPS and IL‐10 + LPS groups, Scale bars, 50 μm. E, Relative ratio of the reticular structure in PBS, LPS and IL‐10 + LPS groups. The rabbit scar models were randomly placed into PBS, LPS and IL‐10 + LPS groups, F, After treatments 0, 3 and 6 weeks, the pictures of scars appearances. G, Masson staining for collagen fibre. H, Collagen index in PBS, LPS and IL‐10 + LPS treatment groups. n = 6 scars in each group. Scale bars, 100 μm, 50 μm. I, Proposed IL‐10 inhibition of LPS‐induced fibrotic mechanisms in human skins

To confirm the effect of IL‐10 on LPS‐induced scar formation *in vitro*, three‐dimensional (3D) culture model was established using embedded NSFs in collagen matrices to generate FPCL in the presence of LPS. In our study model, LPS showed potent contractile effect on FPCL (Figure 7A‐C, **P ˂ .01). IL‐10 treatment group was significantly improved and capable of inhibiting the LPS‐induced FPCL contracture (Figure 7A‐C, *P ˂ .05). Immunohistochemistry results showed that the architecture in PBS group takes the shape of uniform lattice arrangement (Figure 7D). But this architecture was not appeared in LPS group (Figure 7D). In contrast, LPS treatment led to the deposition of collagen in FPCL gel (Figure 7D,E, ***P ˂ .001). Interestingly, the architecture of FPCL was significantly improved in IL‐10 treatment group compared to LPS group (Figure 7D,E, **P ˂ .01). These results confirm that IL‐10 can improve the architecture and inhibit LPS‐induced the contracture of FPCL *in vitro*.

### 3.9 IL‐10 reduces LPS‐induced scar formation in a rabbit ear scar model

To further confirm the effect of IL‐10 on LPS‐induced scar formation *in vivo*, a rabbit ear scar model was established and treated with i.d. injection of PBS, LPS and IL‐10 + LPS. Convincingly, the results showed that the scar appearance in the PBS and IL‐10 + LPS groups was smaller and flatter than in LPS group (Figure 7F). Masson staining revealed that LPS led to a more disordered structure and denser collagen fibre than these observed both in PBS and IL‐10 + LPS groups (Figure 7G,h, *P ˂ .05, **P ˂ .01). These results further confirm that IL‐10 can inhibit LPS‐induced scar formation *in vivo*.

## 4 DISCUSSION

HS results from fibroblastic hyperplasia and is characterized by excessive accumulation of ECM proteins,^9^, ^10^, ^11^, ^12^, ^13^, ^14^, ^45^, ^46^ which complicates wound healing. Although the pathogenesis is unclear, prolonged inflammation is a known contributing factor.^16^, ^36^, ^37^, ^38^, ^47^, ^48^ Our results showed that LPS can enhance ECM proteins synthesis (Figures 2 and 4), FPLC contraction (Figure 7A‐E) and scar formation (Figure 7F‐H). These results suggest a direct role of LPS in scar formation.

Gram‐negative bacteria are common microorganisms in burn wound infections and release endotoxins to the wound surface.^49^, ^50^ LPS is the main component of endotoxin, and its biological activity, cytotoxicity and immunological activity determine the process to a large extent and scar formation. It has been suggested that fibroblasts regulate immune/inflammatory response through TLR4 activated by LPS, leading to NF‐κB activation, cytokine gene transcription and co‐stimulatory molecule expression resulting in inflammation during HS formation.^16^, ^51^, ^52^, ^53^, ^54^ In fact, TLR4 has been shown to be overexpressed at both mRNA and protein levels in HSFs in comparison with NSFs.^16^ The pro‐inflammatory cytokines recruit polymorphonuclear cells, monocytes and macrophages to the wound, thereby producing more pro‐inflammatory cytokines, amplifying the inflammatory response and stimulating scar formation.^55^, ^56^, ^57^, ^58^ Our results confirmed that TLR4 was expressed in dermal fibroblasts (Figures 1,3, 4, 5 and Figure S4). LPS stimulated the expression of TLR4 and pp65 (Figures 3 and 4), increased pro‐inflammatory cytokines (including IFN‐γ, IL‐1β, IL‐6, IL‐8 and MCP‐1, Figure 3) secretion in NSFs, and the ultrastructure of LPS‐stimulated NSFs mimicked their counterparts HSFs (Figure 2). These pro‐inflammatory cytokines then stimulate collagen synthesis and ECM deposition and are also related to other pathological fibrotic disease models.^21^, ^59^, ^60^ Therefore, LPS induces the inversion of NSFs to HSFs and leads to scar formation and skin fibrosis.

IL‐10, an anti‐inflammatory cytokine, has been involved in the attenuating inflammation phase of healing and is known to be elevated in foetal tissues and amniotic fluid.^21^, ^55^, ^61^ Van den Broek^2^ found a decrease in IL‐10 production in HS compared with normal scars. In a study analysing IL‐10 embryonic knockout mice, injury to these mice produced scars not observed in wild‐type counterparts.^29^, ^56^ When IL‐10 was administered to adult mice, a scarless result was obtained.^21^ Interestingly, neonates with a genetic background that lack macrophages and functional neutrophils exhibit normal healing without any obvious scarring.^62^ These results may provide insight to the importance of inflammatory regulation in scar formation. Our results showed that IL‐10 significantly down‐regulated the expression of TLR4 and pp65 (Figures 4 and 5) in HSFs. Convincingly, after IL‐10RA and Cry treatment of IL‐10‐stimulated HSFs, the expression of TLR4 and pp65 was up‐regulated (Figure 5). And the expression change of inflammatory molecules was consistent with those of fibrotic proteins (Figure 6). Combining these findings, these data support the notion that the anti‐inflammatory and anti‐fibrotic function of IL‐10 is promoting the phosphorylation of STAT3 (pSTAT3) through IL‐10R, and regulating TLR4/NF‐κB pathway by the IL‐10R/STAT3 axis in dermal fibroblasts.

After tissue injury, persistent activation of fibroblasts and increased deposition of ECM proteins were usually observed in pathological scars.^63^, ^64^, ^65^ As collagen is one of the key components in ECM, the continuous expression of collagen is an important histological feature to distinguish HS from NS (Figures S1‐S3).^6^, ^66^, ^67^ In addition, activated fibroblast overexpresses α‐SMA, which is a well‐known marker for myofibroblasts and promotes scar contracture.^30^, ^63^, ^68^ Therefore, the regulation of fibroblast excess ECM protein deposition and the transformation to myofibroblasts are the main focus to study the prevention and treatment of HS.

In HS, molecules that regulate intracellular signalling cascades leading to the production of inflammatory mediators are being studied, although pleiotropic mechanisms have been proposed to contribute to the effects of LPS. In this study, we demonstrated that dermal fibroblasts express TLR4 and its intracellular NF‐κB signalling molecules (Figure 1). Our results showed that the expression levels of Col I, Col III and α‐SMA are significantly increased in LPS‐stimulated NSFs (Figures 2,4 and 5) and significantly decreased in IL‐10‐stimulated NSFs (Figures 4 and 6), whereas adding LPS to IL‐10‐stimulated NSFs, these fibrotic proteins were also decreased (Figure 4). In the *in vitro* and *in vivo* models (Figure 7), IL‐10 could improve the architecture and inhibit LPS‐induced FPCL contraction, and these scars displayed a more orderly arrangement, thinner structure and lower collagen index (Figure 7). These results demonstrated that LPS stimulates the expression of pro‐inflammatory cytokines in dermal fibroblasts, and IL‐10 can significantly abrogate the expression of pro‐inflammatory cytokines mediated by LPS‐stimulated NSFs. Therefore, LPS‐stimulated fibroblasts will express pro‐inflammatory cytokines (IFN‐γ, IL‐1β, IL‐6, IL‐8 and MCP‐1), which cause persistent inflammation in injured tissue, thus promoting HS development. Therefore, controlling bacterial contamination and manipulating TLR4 signalling in injured skin fibroblasts may lead to novel strategies for the treatment of HS.

In summary, we have elucidated a novel mechanism for IL‐10 to alleviate LPS‐induced skin scarring. As illustrated schematically in Figure 7I, LPS up‐regulates the expression of inflammatory cytokines, ECM proteins, and the transformation of fibroblasts to myofibroblasts through the TLR4/NF‐κB pathway. Applying of IL‐10RA or Cyr, the expression of inflammatory cytokine is up‐regulated, leading to excess ECM proteins deposition and fibroblasts‐to‐myofibroblasts transformation. We suggest that excess LPS is detrimental to wound healing and ultimately lead to scar tissue formation. Therefore, it is of great significance to understand how natural endotoxin released at the wound microenvironment dictates scar formation. And a down‐regulation of inflammation may be a better option for a suitable scar outcome and improved scar quality.

## CONFLICT OF INTEREST

The authors confirm that there are no conflicts of interest.

## AUTHOR CONTRIBUTION

**Jihong Shi:** Conceptualization (equal); Investigation (equal); Project administration (equal); Writing‐original draft (equal). **Shan Shi:** Data curation (equal); Formal analysis (equal); Methodology (equal). **Wenbo Xie:** Data curation (equal); Formal analysis (lead); Methodology (supporting); Validation (supporting). **Ming Zhao:** Software (supporting); Writing‐original draft (lead); Writing‐review & editing (supporting). **Yan Li:** Data curation (lead); Formal analysis (supporting); Investigation (lead); Methodology (lead). **Jian Zhang:** Data curation (lead); Formal analysis (supporting); Investigation (lead); Methodology (supporting); Validation (supporting). **Na Li:** Data curation (supporting); Resources (lead); Software (supporting); Validation (supporting). **Xiaozhi Bai:** Data curation (supporting); Validation (supporting); Visualization (lead). **Weixia Cai:** Data curation (supporting); Investigation (supporting); Methodology (supporting); Visualization (lead). **Xiaolong Hu:** Formal analysis (lead); Investigation (supporting); Resources (lead); Validation (supporting). **Dahai Hu:** Conceptualization (lead); Project administration (equal); Resources (supporting); Supervision (equal); Writing‐original draft (supporting). **Juntao Han:** Conceptualization (supporting); Resources (supporting); Writing‐original draft (supporting); Writing‐review & editing (supporting). **Hao Guan:** Conceptualization (equal); Funding acquisition (equal); Project administration (equal); Writing‐review & editing (equal).

## Supporting information

Fig S1‐S5
