# Supplementary material for: IL‐10 alleviates lipopolysaccharide‐induced skin scarring via IL‐10R/STAT3 axis regulating TLR4/NF‐κB pathway in dermal fibroblasts
Source: J Cell Mol Med. 2021 Jan 7;25(3):1554–67. doi: 10.1111/jcmm.16250 (PMC7875929; doi:10.1111/jcmm.16250)
Supplement: Supplementary file 1 — Fig S1‐S5 [file JCMM-25-1554-s001.docx]

**Supplementary Material**

**Supplementary Figure 1**


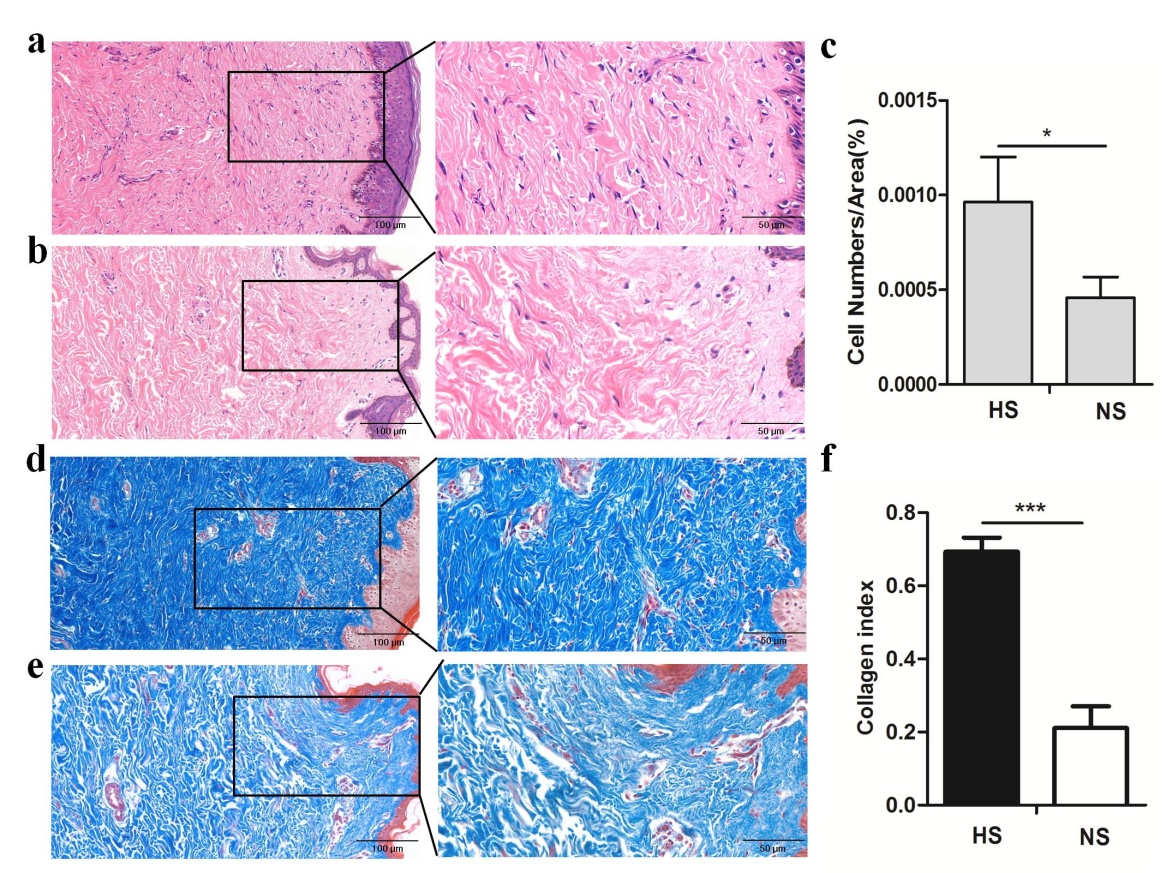


**Supplementary Figure 1** Histology of HS and its counterpart NS stained with HE and Masson. **a** Representative images of HE-stained section of HS group. **b** Representative images of HE-stained section of the counterpart NS group. **c** Differences of fibroblasts in HS and its counterpart NS. **d** Representative images of Masson-stained section of HS group. **e** Representative images of Masson-stained section of the counterpart NS group. **f** Differences of collagen index in HS and its counterpart NS (n = 6; *p < 0.05, ***p < 0.001 compared with its counterpart NS). Scale bars, 100 μm, 50 μm.

**Supplementary Figure 2**


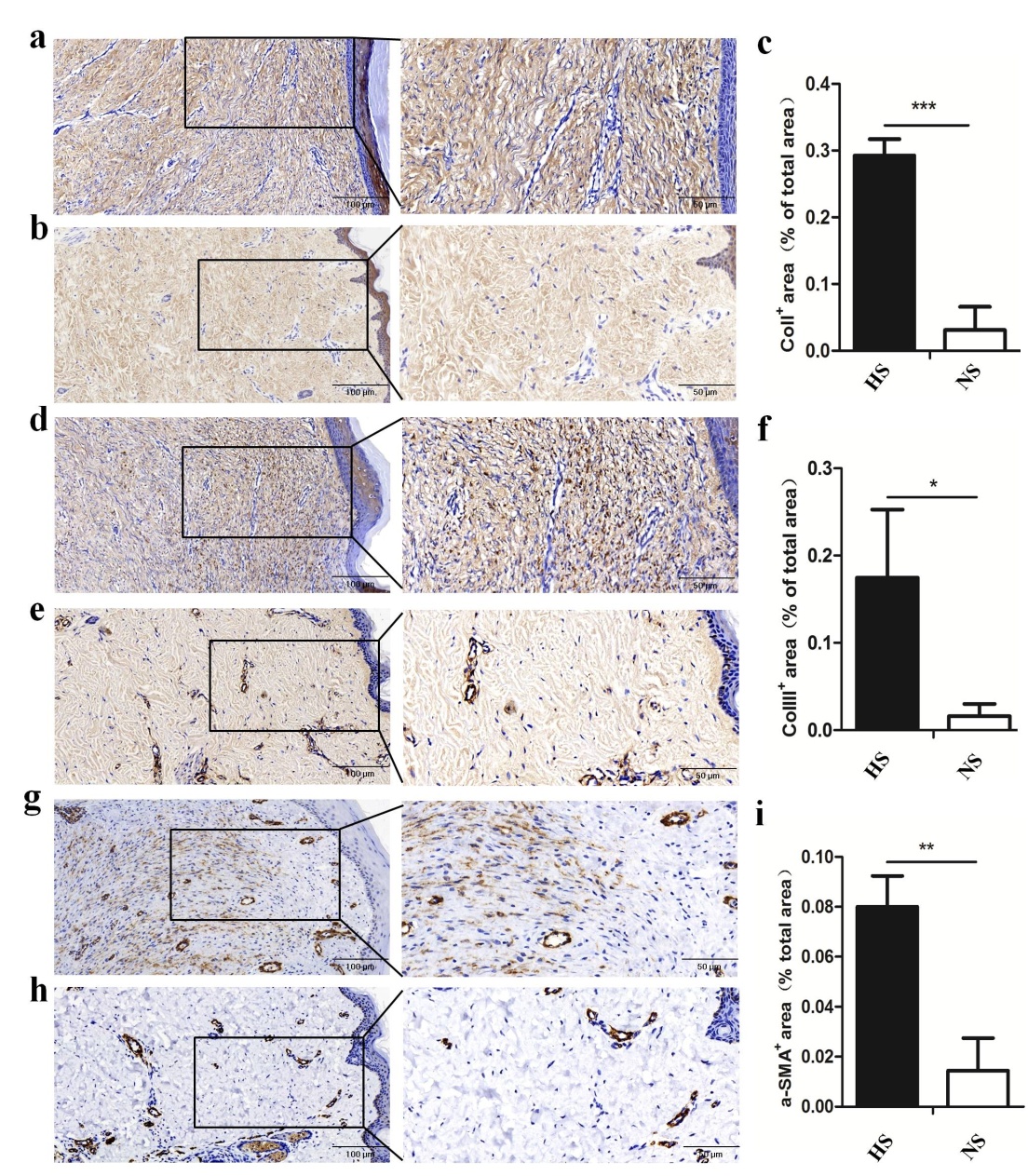


**Supplementary Figure 2** Expression of fibrotic proteins in HS and its counterpart NS. **a** Col I was detected by streptavidin-peroxidase DAB staining of HS. **b** Col I was detected by streptavidin-peroxidase DAB staining of the counterpart NS. **c** Differentiation of Col I positive area in HS and its counterpart NS. **d** Col III was detected by streptavidin-peroxidase DAB staining of HS. **e** Col III was detected by streptavidin-peroxidase DAB staining of the counterpart NS. **f** Differentiation of Col III positive area in HS and its counterpart NS. **g** α-SMA was detected by streptavidin-peroxidase DAB staining of HS. **h** α-SMA was detected by streptavidin-peroxidase DAB staining of the counterpart NS. **i** Differentiation of α-SMA positive fibroblasts in HS and its counterpart NS (n = 6; *p < 0.05, **p < 0.01, ***p < 0.001 compared with its counterpart NS ). Scale bars, 100 μm, 50 μm.

**Supplementary Figure 3**

**
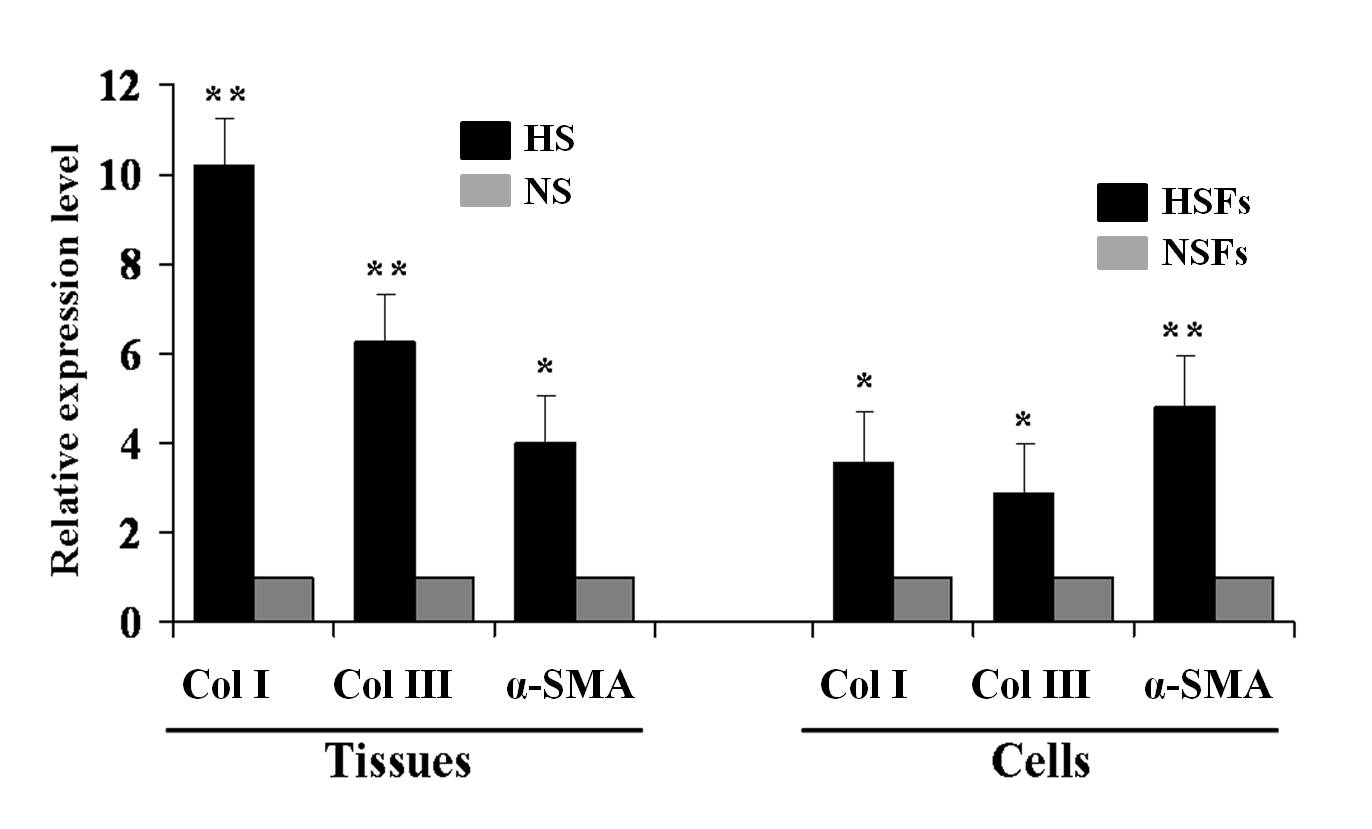
**

**Supplementary Figure 3** The differential transcription levels of Col I, Col III, and α-SMA in HS/NS and their counterpart HSFs/NSFs. PCR products were measured by the threshold cycle (Ct) at which specific fluorescence became detectable. The Ct was used for kinetic analysis and was proportional to the initial number of target copies in the sample. The mRNA levels of each gene were normalized to the housekeeping gene encoding GAPDH. HSs and their counterpart NSs were collected, and HSFs/NSFs were cultured in DMEM for 24 h. The mRNA levels of Col I, Col III, and α-SMA were extracted and analyzed by RT-qPCR. Data are expressed as the mean ± SEM; n = 3; *p < 0.05, **p < 0.01 compared with the corresponding controls.

**Supplementary Figure 4**


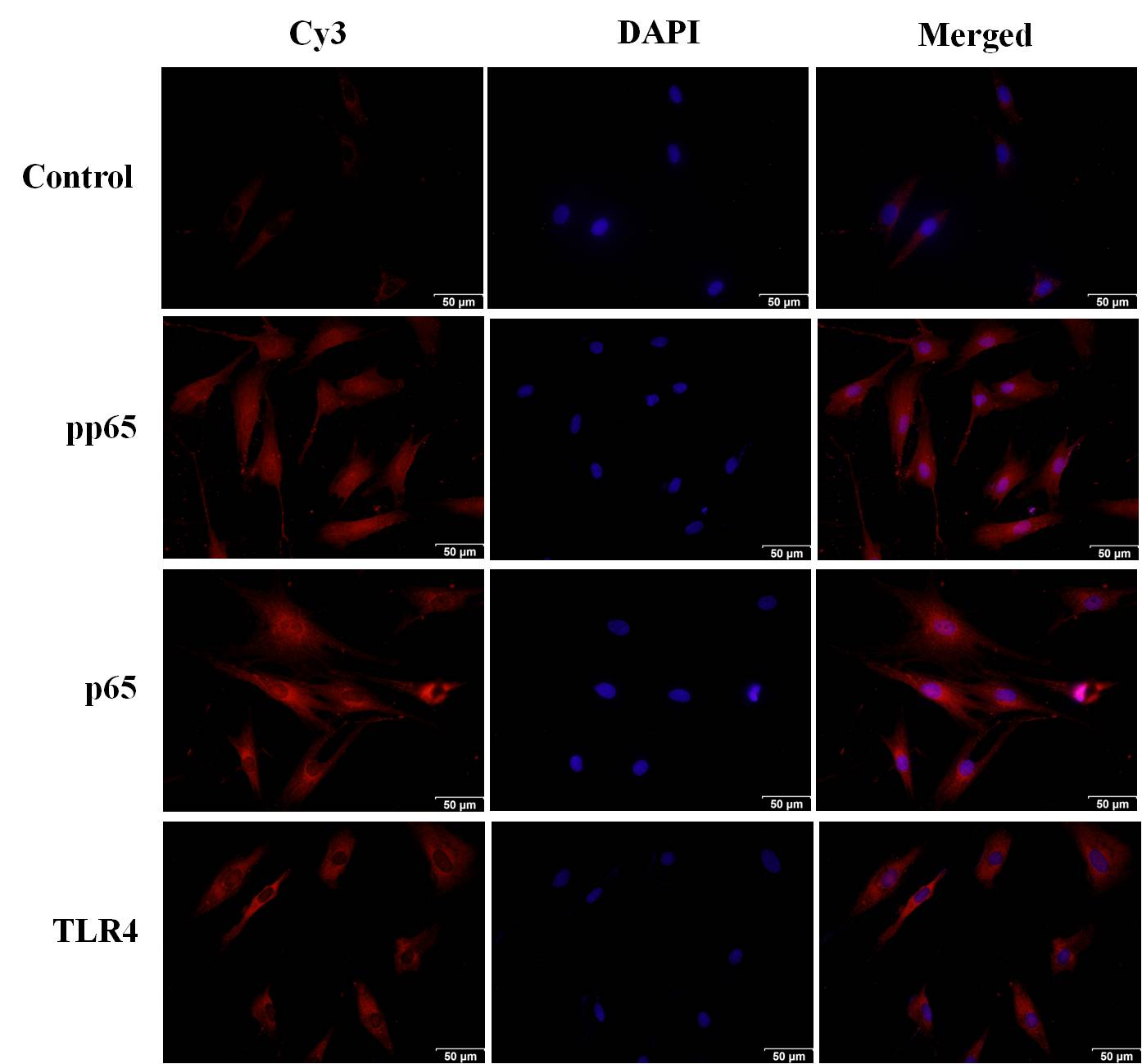


**Supplementary Figure 4** Inflammatory molecules expression in HSFs. HSFs were cultured on cover slips, and grown to 50% confluence, fixed with paraformaldehyde. The expression of pp65, p65 and TLR4 was founded using their specific mAbs and a Cy3-conjugated secondary antibody, fibroblasts nuclei with DAPI, by fluorescence microscope in the slide at three randomly chosen regions (n = 3). Scale bars, 50 μm.

**Supplementary Figure 5**

**
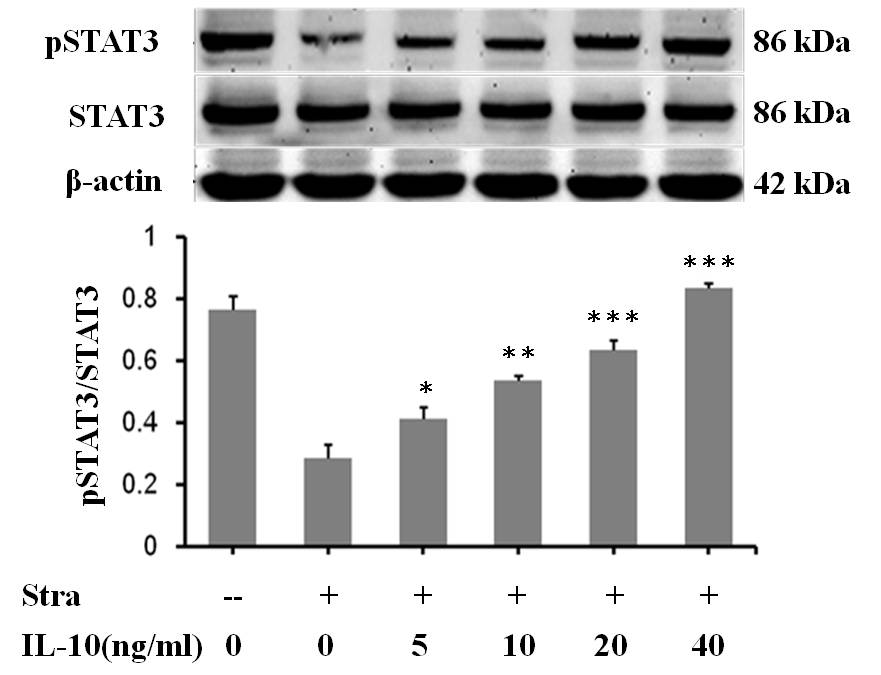
**

**Supplementary Figure 5** IL-10-mediated activation of pSTAT3 in HSFs. HSFs with 70-80% confluent were starved by culturing in serum-depleted medium for 12-16 h before exposure to different concentrations of IL-10 for 30 min. pSTAT3 and STAT3 protein expression and changes in the pSTAT3/STAT3 ratio. Data expressed as the mean ± SEM; n = 3, *p ˂ 0.05, **p ˂ 0.01, ***p ˂ 0.001 compared with the starvation control.
